# A German’s Experience with Quacks

**Published:** 1876-04

**Authors:** 


### A GERMAN'S EXPERIENCE WITH QUACKS.

The willingness of the public to be deceived by, and the alacrity even, with which they patronize traveling mountebanks, who parade the declaration of their accomplishments from the bill-boards and brick walls of the city, or through the flaming hand-bill thrust in at your door, and whose own assurances are the only warrant of their skill, and who, when their ignorance and failures become too glaringly evident in one place, flee to another, is a constant wonder and surprise to us.

No matter how absurd their professions, or ridiculous their pretentions, willing and even anxious dupes, await their coming with a longing and faith that never is satiated. Be the charlatan a German, with unpronounceable name, cunning of tongue, urbane of manner, and lavish of printers' ink; an ignorant tape-worm doctor, with liveried lackey, and jars of worms for the public inspection; an "Indian doctor" with his roots and herbs mysteriously compounded, and suited to all ailments; or the clarivoyant, who prescribes through some defunct old chap who lived in an age when medical knowledge was of the crudest character—all, all will entice the unsophisticated, and what is stranger still, intelligent people, to their doors.

Recently, we enjoyed a call from a good natured German, from Snyder county, Pennsylvania. He had been, and is now, a great sufferer from catarrh, and had patronized every traveling doctor that came within his reach, with a pertinacity and confidence in final success in securing relief from his malady, worthy to be classed with the efforts of any martyr of which history has given us information.

He was seated in our private office, and for two mortal hours, gave us such a recital of his experience with doctors and medicines that was well worth the time devoted to the listening.

Said he, in the funniest of English, "When I got dem catarrh on my nose, I went to see one great Injun doctor, what stopped at our town—he said I had de *dry* catarrh, when I told him all de while dot it was *wet* wid me, ober he said dot makes no difference, und he scharged me twelf dollar for tree gallons of sirupe. I took him all up, but vas no better, und I tolt him so, und he sait I must take six gallon more. I tinks dot wun big tose, ober I took him, cause he sait dot would cure me *sure*. But when dot was *all*, I went to him again, on de next stot, und when I tolled him I was not besser, he says vel, I be a tam fool, und nobody could cure me, und I tought so too! Den I went purty soon, to anoder doctor at Sunbury. He was a great English doctor, what come all de way from Paris, or some of dem blaces. He vas over 80 years olt, und his hair vas white as snow. Now, tinks I, if dis doctor isn't honest, where will I find um. So I gif him twenty-five dollar, und he put some—what you call spanish flies—in my ear, und de next morning you youst would not have knowd me from any odder feller, because I didn't know myself. My het was all shvelled up, und I couldn't hear notting, und I vas in such bain dot I tought I would die. But de old doctor write on a slait, it was all right, und I tought so too! Well, by und by, my ear—one of 'em broket, und when I hold my nose und blow, I can squirt water, blood und stuff out of dot, youst about a raut--stop, I'll show you—(and he did), und now I hears notting at all from dot ears. Den he sait he would put some more in, und I sait I guess not. Den he say well, I gif you some medicine for your nose, und I say what it is, more spanish flies? und he say no, it be sweet oil und morphy, und I must put him way up my nose wid a fedder. Well, I goes hame mit dis stuff und pulls a fedder from de old goose, sticks him in de oil und runs him up my nose, as de old doctor sait; und when I did dot youst once, I schneezed, und schneezed, *und* I schneezed, undil I couldn't schneeze no more, und den I lait down on de floor und schneezed some more. Und den I went over to Sunbury to pour de whole bottle full in dot old feller's nose, und *he* was gone too, just like dat odder fellers.

"Und den cum by und by, one dem fellers what you call a clerry-voyance—he goes into fitz you know, und den tells what he sees in a feller's insides by looking like one tam idiots at his outsides. Well, I pait him four dollar to make dem faces at me, und he sait I had a tape-worms. I askt him how de deyful dot tape-worms got in my het, und he sait up fro de tupes in my troat, und I tinks, by jimmeny, ish dot so, und askt him whot does tape-worms cost, und he sait twenty-five dollar. I went home so quick as I could und got de moneyes, und took dot medicines for de tape-worms. I tought I should youst vomit my outsides in, und my pelly it gramped und ached, und—well, I was youst awful sick, und den dot clerry-voyance chap he tolt me dot tape-worms vas all chopped up fine, und I could not see him, ober he vas dare shure. Und den I tinks to myself if dot medicines chops up dot tape-worms fine, what der tyful would it do to my stummicks und all dem odder internal arrangements; und den purty soon, I tinks I vas 'solt,' und I goes home to dinks about it. De next week my het vas so bad I goes back to dot fitz feller, und den de tavern keeper tolt me he had gone youst like all dem udder fellers, und he hot my twenty-five dollar und I hat my het youst de same as pefore, all de vile!"

And in this manner our German friend related one experience after another, until our sides ached with the laughter that his odd expressions and queer experiences provoked. Finally, he said: "Now, Doctor, I tells you youst vat I tinks—I tinks doctors all one tam humbugs, what *you* tinks, too?" And we agreed with him.

